# What Is the Most Effective Frictionless Method for Retracting Anterior Teeth When Using Buccal Fixed-Appliance Therapy? A Systematic Review

**DOI:** 10.3390/jcm13010231

**Published:** 2023-12-30

**Authors:** Mohammad Naem Kheshfeh, Mohammad Younis Hajeer, Mhd. Firas Al Hinnawi, Mohammed Adel Awawdeh, Farraj Albalawi, Ghada Serhan Alotaib, Mohammad Khursheed Alam, Ahmad Salim Zakaria

**Affiliations:** 1 Department of Orthodontics, Faculty of Dentistry, University of Damascus, Damascus P.O. Box 16046, Syria; mohammad.kheshfeh@damascusuniversity.edu.sy; 2 Biomedical Engineering Department, Faculty of Electrical and Mechanical Engineering, University of Damascus, Damascus P.O. Box 16046, Syria; firas.alhinnawi@damascusuniversity.edu.sy; 3 Preventive Dental Science Department, College of Dentistry, King Saud bin Abdulaziz University for Health Sciences (KSAU-HS), Riyadh 11426, Saudi Arabia; balawif@ksau-hs.edu.sa (F.A.); alotaibigh@mngha.med.sa (G.S.A.); 4 King Abdullah International Medical Research Center, Ministry of National Guard Health Affairs, Riyadh 11481, Saudi Arabia; 5 Dental Services King Abdulaziz Medical City, Ministry of the National Guard-Health Affairs, Riyadh 11426, Saudi Arabia; 6 College of Medicine & Dentistry, Ulster University, Birmingham B4 6BN, UK; 7 Orthodontic Division, Preventive Dentistry Department, College of Dentistry, Jouf University, Sakaka 72345, Saudi Arabia; mkalam@ju.edu.sa; 8 Department of Dental Research Cell, Saveetha Institute of Medical and Technical Sciences, Saveetha Dental College and Hospitals, Chennai 600077, India; 9 Department of Public Health, Faculty of Allied Health Sciences, Daffodil International University, Dhaka 1207, Bangladesh; 10 Department of Orthodontics, School of Dental Sciences, Universiti Sains Malaysia, Penang 11700, Kelantan, Malaysia; ahmadzakaria@student.usm.my

**Keywords:** frictionless techniques, retraction springs, adult, anterior teeth retraction

## Abstract

There are various techniques and designs for springs used in orthodontic treatment, including frictionless methods for closing spaces. However, there is limited explicit evidence to support the superiority of one method over another. This review aims to investigate the available evidence and highlight the advantages of these different methods. This review contained six papers, and information such as study design, spring design, applied force systems, variables studied, follow-up period, and records were extracted. All of the studies focused on canine retraction with the Ladanyi spring showing the highest rate of movement (1.8 mm per month) among all springs for upper canine retraction. The Gjessing and T-loop springs outperformed the Reverse Closing Loop and Ricketts spring, respectively, substantially. In terms of tip control, the T-loop spring showed a clear advantage over the modified Marcotte spring with a difference of 1.2° vs. 6.6° per 3 months. Additionally, it was observed that the Reverse Closing Loop caused a significant loss of anchorage during canine retraction with a medial movement of 2.4 mm. When comparing wire types, no significant differences were found between TMA and Nitinol, while stainless steel was found to be less effective in terms of movement rate and tip control. However, the results indicated that there was no clear evidence that one specific technique was definitively preferable to another; therefore, there is an urgent need for more studies with proper study designs to produce more robust conclusions.

## 1. Introduction

The orthodontic camouflage plan is a commonly used treatment option for managing class II cases. This involved extracting the first premolars to lessen the problem in the sagittal plane [1], followed by a retraction that could be completed in two phases [2], or in one phase, which is known as an en masse retraction [3]. Cases involving the retraction of the anterior teeth make up a significant portion of the cases seen by orthodontists daily [1]. The main objective during this stage is to achieve anterior teeth retraction with the best possible control, as moving the anterior teeth requires a lot of time out of the overall orthodontic treatment [4].

Two techniques can be used to retract the anterior teeth: sliding or frictionless. In the frictionless technique, the first step of the two-step retraction technique involves the backward movement of the canine using a loop-based sectional archwire originating from the first molars on either side of the dental arch [5]. In the second stage, incisors can be retracted using a utility arch [6] or a continuous archwire augmented with T-shaped or inverted-L-shaped loops [7]. En-mass retraction can be performed using loop-based continuous archwires that can be activated to retract the whole block of teeth backward [7].

Techniques that use loops functioning as springs to move a single tooth, like a canine, or a group of teeth, such four upper or lower incisors, are referred to as “frictionless movement”. Numerous examples, including Burstone’s T-loop, Ricketts spring, and Gjessing’s spring, may be found in the orthodontic literature [5,8,9]. Some studies assert that sectional archwires with contained loops are superior to other methods for controlling teeth movement in the three-dimensional location of the canine. It has been suggested that the use of sectional archwires can prevent the bite from deepening, resolve friction problems, and accelerate movement [9,10]. When it comes to retracting anterior teeth en masse, several clinical reports have demonstrated that sectional techniques offer superior control over tooth movement and are more effective when executed with precision. This is due to the fact that working with these techniques requires a thorough understanding and precise control over the generated force systems [11]. The force system created by the activated spring is largely determined by the placement of the T-loop on the arch [11].

The springs used in loop-based techniques differ in their design and methods of activation, and the practitioner is confused about which spring is the most optimal. Some dental movements may prefer one spring over another; one study showed that moving the canine using a T-loop spring was more effective in the anterior–posterior direction than the Rickett’s retractor, while the Rickett’s retractor had the advantage in the vertical direction [5]. After excavating the orthodontic literature, no published systematic review was found that compared several designs of loop-based techniques in the retraction of upper anterior teeth. Since there was no previous systematic review that examined this topic and these comparisons, it was intended to conduct this review to answer the following two related focused review questions: In the presence of several loop-based techniques in anterior teeth retraction, what is the most effective technique?

## 2. Materials and Methods

This systematic review has been submitted in the PROSPERO database (registration number CRD42023452266) in compliance with the 2020 Preferred Reporting Items for Systematic Reviews (PRISMA) standards, as of 17 August 2023.

### 2.1. Eligibility Criteria

Based on the PICOS framework, inclusion and exclusion criteria were developed. Patients who require extraction of the first premolars and retraction of the anterior teeth due to any type of malocclusion were the target population. The intervention was any type of frictionless method for retraction. The comparison was any other form of retraction springs with a frictionless technique. The rate of orthodontic tooth movement, inclination (torque), root resorption, anchoring loss, and control of angulation (tipping) were the main areas of interest in this study. Studies were excluded when the study (a) employed magnetic elements for retraction; (b) included craniofacial anomalies; (c) used any surgical or non-surgical means to accelerate dental movement; (d) involved animals. All of the included papers (RCTs and non-RCTs) were published in English and were either split-mouth or parallel-group clinical investigations.

### 2.2. Search Strategy

For every research paper reported up to 31 July 2023, an electronic research review was conducted using the following databases: PubMed^®^, Scopus^®^, EMBASE^®^, the Cochrane Central Register of Controlled Trials, Web of Science™, and Google™ Scholar.

Keywords are presented in Table 1, and details related to the search strategy are presented in Table A1.

### 2.3. Study Selection and Data Extraction

The two writers conducting the review (MNK and MYH) followed specific inclusion criteria when selecting studies. In cases where there was a disagreement, the third writer (MAA) was consulted to resolve it. If there were any questions or need for clarification, the authors of the retrieved publications were contacted. Firstly, the title and abstract of each article were used to filter them. The full text of each chosen article was then evaluated in the next stage. Only articles that met at least one of the qualifying requirements were included in the review. The final selection of articles all met the predetermined criteria. Information such as the authors’ names, research design, sample size, mean patient age, retraction force application technique and intensity, observation duration, follow-up data, and outcome measures were all extracted from the papers.

### 2.4. Assessment of Risk of Bias in Individual Studies

The authors used Cochrane’s Risk of Bias tool for RCTs to assess (high, low, or unclear) from five domains for individual items (selection, performance, attrition, reporting, and other) [12]. For each study, the overall risk of bias was calculated. When all fields showed a low risk of bias, it was deemed that the risk of bias was low. When one or more fields showed an unclear or high risk of bias, respectively, the conditions were deemed unclear or high. The seven domains of the ROBINS-I instrument were used in non-randomized experiments (bias due to confounding, bias in selection of participants into the study, bias in classification of interventions, bias due to deviations from intended interventions, bias due to missing data, bias in measurement of outcomes, and bias in selection of the reported result) were used to assess individual items (low, moderate, serious, critical, or no information) [13]. For individual studies, the overall risk of bias was also evaluated.

## 3. Results

### 3.1. Literature Search Flow and the Retrieved Studies

**Figure 1:**
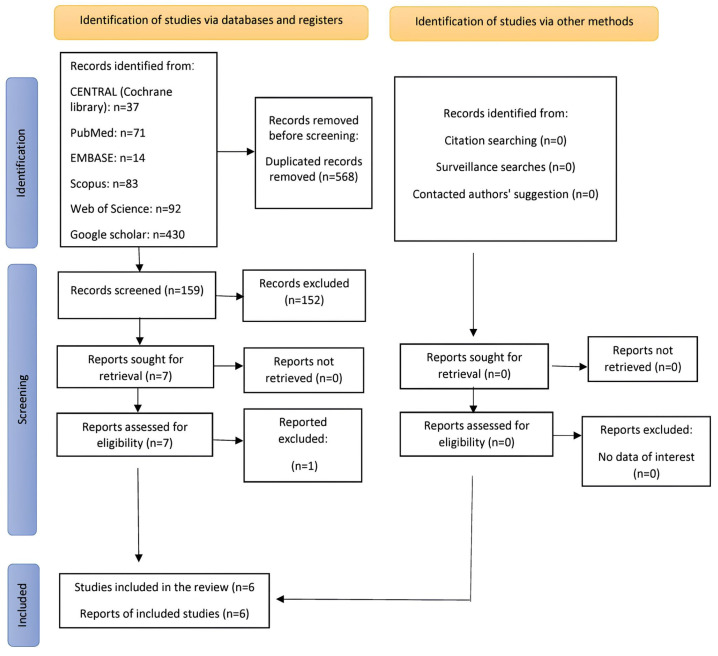
Study identification, screening, and inclusion PRISMA flow diagram.

Using an electronic search, 536 studies were located. After eliminating the duplicates, 159 articles were thoroughly examined. To find publications that met the inclusion requirements, the titles, abstracts, and full texts of the articles were screened. Articles that did not fit these requirements were all disqualified. Six publications in total were included in the systematic review [5,14,15,16,17,18]. Figure 1 shows the PRISMA flow diagram for research identification, screening, and inclusion.

### 3.2. Characteristics of the Included Studies

Table 2 displays the characteristics of the six studies that were included. Three of the studies were randomized split-mouth clinical trial designs [14,15,16], two were non-randomized split-mouth designs [17,18], and one was a non-randomized two-group comparative study [5]. The studies included 130 adult patients. Two studies (33%) reported the gender distribution within the sample; the male-to-female ratio was approximately 1:1 in the two studies [15,16]. The ages of the study’s sample were mentioned in five (83%) of the research with the enrolled samples’ mean ages ranging from 11 to 23 years [5,14,15,16,17].

All six comparative studies evaluated the retraction of the upper canines, and one of these examined the retraction of the lower canines in addition to the upper canines [17]. These studies involved the comparison of two different methods of sectional archwires [5,14,15,16,17,18].

There were variations in the bracket prescriptions among the studies. Four of the six studies used brackets with a slot height of 0.018 inches [15,16,17,18], two used brackets with the MBT prescription [16,18], one used brackets with the Alexander prescription [15], and one used brackets without angulation or torque [17]. One study used brackets with a slot height of 0.022 inches using the MBT prescription, and one study used a combination of the two types [19].

The canine retraction spring designs differed, as the Gjessing retraction spring was employed and its efficacy was contrasted with the Reverse Closing Loop [17]. The retraction efficacy was compared using T-loop springs versus a modified Marcotte spring [14] and Ricketts maxillary canine retractor [5]. T-loop springs were compared with each other by comparing two different materials (TMA versus nitinol [15] and TMA versus stainless steel [18]). A study compared the effectiveness of the retraction between the Ladanyi spring and the reverse closing loop [16].

All included studies evaluated the canine retraction rate and the change in the canine tip (angulation) following retraction [5,14,15,16,17,18]. Of the included research, four studies (67% of the total) examined anchoring loss during canine retraction [5,14,16,17], and four research studies (67% of all included papers) looked into the canine’s rotational movements during retraction [5,14,16,18].

Several evaluation instruments were employed to investigate the variables. Multiple measurement tools were used in three trials [14,16,18]. Dental casts were scanned or photographed and then placed into software for analysis in four research (67% of the total included studies) to examine some of the variables [14,15,16,18]. One study (17% of all included studies) used an intraoral acrylic splint with embedded hooks to calculate linear measurements using a digital caliper, and the measurements were made intraorally [18].

Four studies (67% of all included studies) used lateral cephalometrics to study some of the variables, including variables the evaluation of canine distalization rate [16,17], anchorage loss [16,17], and tipping magnitude [14,17,18].

One study used cone-beam computed tomography (CBCT) to study some of the variables, such as evaluating the amount of canine retraction, canine tipping, canine rotation, canine torque, root resorption of canines, and anchorage loss control [5].

### 3.3. Risk of Bias of Included Studies

**Figure 2:**
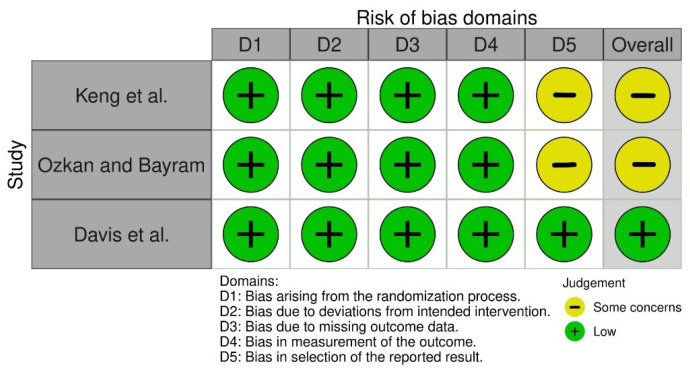
Risk of bias of the RCTs that were included [14,15,16].

**Figure 3:**
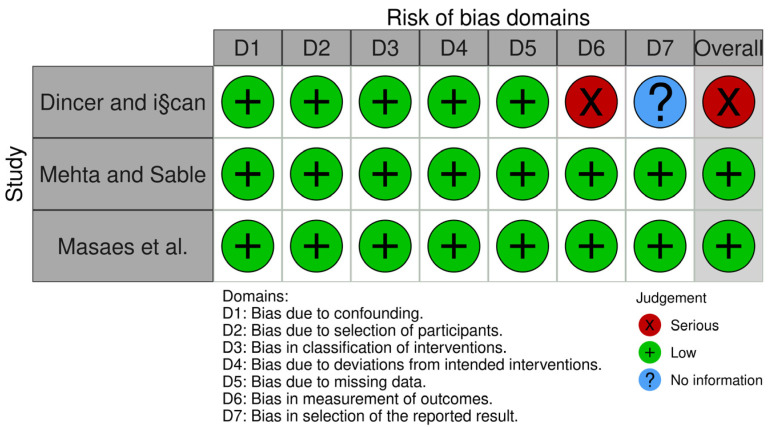
Risk of bias of the comparative non-randomized studies that were included [5,17,18].

One randomized trial was deemed to have a low risk of bias [14], and two were deemed to be of some concern because there were not enough data to challenge the results’ selectivity [15,16]. Of the non-randomized trials, two were classified as low risk of bias [5,18], and one was classified as having a serious risk of bias [17]. Figure 2 and Figure 3 and Table A1 summarize the overall risk of bias in the included studies and the reasons beyond each judgement.

### 3.4. Effects of Intervention

All available studies investigated only partial canine retraction among the components of space closure in orthodontic practice. The results of these studies are summarized in Table 3. Six trials evaluated the outcome between different methods of loop-based techniques [5,14,15,16,17,18]. The results of these studies were not quantitatively synthesized due to the different methods used in the retraction.

#### 3.4.1. Rate of Canine Retraction

##### According to the Details of the Wires

The 0.018 × 0.025-inch nickel–titanium spring was similar to that of a 0.017 × 0.025-inch TMA spring [15], but the 0.017 × 0.025-inch nickel–titanium spring had a greater retraction rate than the 0.016 × 0.022 stainless steel spring with a mean retraction rate of 1.36 mm/month vs. 1.05 mm/month, respectively [18].

##### According to the Spring Designs

Gjessing’s retraction spring led to a significantly greater canine retraction rate compared to the Reverse Closing Loop in one study (upper canines: a mean of 0.85 mm/month and 0.59 mm/month for both techniques, respectively; lower canines: 1.03 mm/month and 0.39 mm/month for both techniques, respectively) [17]. However, using a reverse closing loop similar to that of the Ladanyi spring [16], the retraction using the modified Marcotte spring had a significantly greater mean rate than the T-loop spring with a mean of 1.18 mm/month and 0.7 mm/month, respectively [14]. Furthermore, the retraction rate with the T-loop spring was significantly greater than the Ricketts canine retraction spring with a mean of 1.85 mm/month and 1.10 mm/month, respectively [5].

#### 3.4.2. Canine Tipping and Rotation following Canine Retraction

There were no significant differences in tipping change between the reverse closing loop versus Gjessing’s retraction spring, TMA T-loop versus nitinol T-loop, the Reverse Closing Loop versus Ladanyi spring, and the T-loop spring versus Ricketts maxillary canine retractor [5,15,16,17]. The 0.017 × 0.025-inch TMA T-loop spring had better tip control than 0.016 × 0.022-inch stainless steel T-loop with a mean of 7.8 degrees/4 months and 10 degrees/4 months, respectively [18]. The 0.017 × 0.025-inch TMA T-loop spring had better tipping control than the modified 0.017 × 0.025-inch TMA Marcotte’s spring with a mean of 1.2 degrees/3 months and 6.6 degrees/3 months, respectively [14].

Four trials investigated rotation control [5,14,16,18], and two found significant differences. The stainless steel T-loop spring offered better rotational control (the ratio of how much the canine angle changed to its angle before the movement was 39.44%) over the TMA T-loop (50.82%) [18]. The retraction using a 0.017 × 0.025-inch TMA T-loop spring offered better rotation control than a Ricketts maxillary canine retractor formed by 0.016 × 0.016-inch blue Elgiloy wire (a mean of 5.7 degrees vs. 14.9 degrees, respectively) [5].

#### 3.4.3. Anchorage Loss following Canine Retraction

According to the spring designs, four trials evaluated this outcome between different methods of loop-based techniques. There were no significant differences in anchorage loss between the reversed closing loop versus the Ladanyi spring and the T-loop spring versus Ricketts maxillary canine retractor [5,16]. However, anchorage loss when using the reversed closing loop was greater than when using Gjessing’s retraction spring (a mean of 2.46 mm vs. 1.63 mm, respectively) [17]. The anchorage loss upon retraction using the modified Marcotte spring was significantly greater than the T-loop spring (a mean of 0.97 mm vs. 0.25 mm, respectively) [14].

## 4. Discussion

This is the first systematic review comparing the effectiveness of the different types of frictionless methods for retracting the canine, incisors, or the six upper anterior teeth. The evidence and data supporting the superiority of one retraction technique or method over another was insufficient due to a lack of research comparing the differences between the various approaches.

### 4.1. Rate of Canine Retraction

The retraction rate of 0.017 × 0.025-inch TMA T-loop springs was greater than that of 0.016 × 0.022 stainless steel T-loop springs [18]. This can be explained by their high elasticity, more stable forces, and high M/F ratio when using TMA wire. Gjessing’s and modified Marcotte springs resulted in significantly greater canine retraction rates compared to reverse closing loop and T-loop springs, respectively [14,17]. The possible reason for this could be the stimulation of the loops by closing them (toward the original bending) in Gjessing and modified Marcotte springs, which is more effective than stimulating by opening them, as is the case with T-loop springs.

The retraction rate with the TMA T-loop spring was significantly greater than the blue Elgiloy Ricketts canine retraction spring [5]; the possible reason for this could be due to the characteristics of the cobalt–chromium alloy (blue Elgiloy), which excels in its ability to formability beta-titanium alloy (TMA). The high formability of blue Elgiloy means that the Ricketts spring may be exposed to more residual moment reductions during the deactivation. In contrast, the TMA T-loop spring shows a more stable moment due to the strength of the wire and the softness curvature bends.

According to the force magnitude, the force required to retract the canines varied between 120 g [16], 150 g [14,15,17], and 200 g [18]. In Masaes’s study, the initial activation force for a T-loop spring was measured at 344 g [5]. It was observed that the smaller force field (120–150 g) resulted in a higher rate of movement, indicating that tooth movement occurs through direct bone resorption, leading to faster tooth movement.

### 4.2. Canine Tipping and Rotation following Canine Retraction

The retraction by a 0.016 × 0.022 stainless steel T-loop spring caused a tipping greater than the 0.017 × 0.025-inch TMA T-loop spring [18]; this can be justified by the high hardness of the stainless steel wire, which did not allow for achieving the desired M/F ratio for uprighting the root. The T-loop spring caused greater tipping than Marcotte’s spring [14]; this may be explained by the T-loop spring having pre-activation bends that help promote bodily movement. The retraction of the stainless steel T-loop ensures higher control over the rotation than the TMA T-loop spring [18]. This rotation was less likely due to the higher hardness of the stainless-steel wire.

### 4.3. Anchorage Loss following Canine Retraction

The Gjessing spring provided less anchorage loss than the reversed closing loop [17], which may be due to the presence of a proportional β moment by the distal leg of the Gjessing spring, providing less mesial movement of the molars. The T-loop spring provided less anchorage loss than the Marcotte spring [14], which may be attributed to the design of the T-loop spring, which included pre-activation bends evenly located in both arms of the spring, whereas the Marcotte spring only included an anti-extrusion bend in the distal arm

### 4.4. Limitations

The lack of homogeneity of comparisons between studies was a limitation of this review, as this prevented the possibility of conducting a meta-analysis. The number of studies that have investigated this topic of practice was very small, especially since only three out of the six studies were randomized controlled trials that investigated this main topic in orthodontist practice.

## 5. Conclusions

A Gjessing spring provided a higher rate of retraction and greater tip control than a reversed closing loop, while there were no differences between the effectiveness of the reversed closing loop and the Ladanyi spring. The modified Marcotte spring outperformed the T-loop spring in rate of movement, while the T-loop spring outperformed the Ricketts spring in terms of tip control. There were no differences in terms of effectiveness between TMA and Nitinol springs, while TMA was superior to stainless steel in terms of rate of movement and tip control. However, based on the small number of studies that discussed the comparison of different methods of retraction, the evidence was insufficient to issue strong indications, so there is a need to conduct more studies in this field of orthodontics.

## Figures and Tables

**Table 1:** Keywords used in the search.

Orthodontics	Class II relationship, extraction of the first premolars, severe crowding, maxillary dentoalveolar protrusion, bimaxillary protrusion, permanent occlusion, and anterior open bite.
Treatment plan	Upper anterior teeth retraction, lower anterior teeth retraction, space closure, incisors retraction, canine retraction, en masse retraction, moving anterior teeth backward.
Outcomes	Anchorage loss, rotation, inclination, torque, angulation, tipping, and root resorption are among the factors that affect orthodontic tooth movement rate, amount, speed, velocity, and duration.
Intervention	Frictionless mechanics, sectional technique, segmental technique, springs for retracting T-loop, L-loop, Ricketts spring, Marcotte spring, Ladanyi spring, Gjessing retraction spring, and Reverse Closing Loop.

**Table 2:** Characteristics of the included trials.

Authors	Patient Count, Average Age, and Study Design	The Application Mechanism and Force Intensity	Outcomes	Follow-Up Period	Extraction Time/Anchorage	Evaluation Instrument
Dincer and Iscan, 1994 [17]	Upper Jaw: 12 Range: 11 y, 9 m to 19 y, 9 m; mean age: 15 y. Lower Jaw: 8 Range: 11 y, 5 m to 16 y, 10 m Mean age: 13 y, 7 m. Split-mouth	Group 1: S.S 0.016 × 0.022-inch Reverse Closing Loop Group 2: S.S 0.016 × 0.022-inch Gjessing’s retraction spring Force: 150 g Bracket: 0.018 in/without angulation or torque.	Canine retraction rate, treatment time, tipping, and anchorage loss in both jaws	Until completion of the retraction of the canine	Immediately before retraction	CRs before and at the end of canine retraction Eight linear and two angular measurements were made on the cephalometric tracings for each upper and lower treatment group
Keng et al., 2012 [15]	12 (6 male, 6 female) Age: 13 y, 3 m to 20 y, 1 m. Median: 14 y, 4 m both parallel and split-mouth	Group 1: TMA 0.017 × 0.025 TLS Group 2: NITI 0.018 × 0.025 TLS Force: 150 g Bracket: 0.018 in/the Wick Alexander prescription.	Rate of space closure per month and changes in upper canine tipping	Until completion of the canine retraction or achieving class I relationships.	Class II elastics, Nance and TADs according to the requirements of anchorage for each case	Upper dental impressions at the start and at each visit to assess space closure. A digital image of each cast was taken
Mehta and Sable, 2013 [18]	15 Split-mouth	Group 1: TMA 0.017 × 0.025 TLS Group 2: S.S 0.016 × 0.022 TLS Force: 200 g Bracket: 0.018 in/MBT.	Amount of maxillary canine retraction, tipping, and rotation control	4-month period	At the beginning of treatment. Nance holding arch	The rotation of the canines during retraction was evaluated by making occlusal photographs of the study models at the study’s start (T0) and end (T4). CRs at (T0) and (T4) for angulation. Linear measurement with a digital caliper
Ozkan and Bayram, 2016 [16]	36 (17 male, 19 female) Mean age 16.8 ± 2.4 y Split-mouth	Group 1: Remaloy wires 0.016 × 0.022 Reverse Closing Loop Group 2: Remaloy wires 0.016 × 0.022 Ladanyi spring to the other canine randomly Force: 120 to 150 g Bracket: 0.018 in/MBT.	Canine distalization rates, anchorage loss, and rotation	Prior to acquiring a class I canine relationship	At the beginning of treatment. Indirect skeletal anchorage system: mini-implant-supported Nance appliance. Direct skeletal anchorage system: mini-implant	Casts before and after retraction for rotation study. The mesial and distal contact points of the canines were marked on the dental casts and transferred to the computer via a scanner CRs before the retraction and when the canines reached the class 1 relationship
Davis et al., 2019 [14]	24 13–20 y Split-mouth	Group 1: 0.017 × 0.025 modified Marcotte spring Group 2: TLS Force: 150 g Bracket: 0.022-in/MBT.	Amount of retraction, rate of retraction, anchorage loss, tipping, and rotation	To completion of 18 weeks	At the beginning of treatment. TPA and banding of second molars	Study models at the beginning and after 18 weeks. The casts were then scanned CRS uses radiopaque TMA wire markers to differentiate the right and left sides for tipping
Masaes et al., 2022 [5]	2 group (31) 14–23 y Group 1: n: 14 by T-loop spring (2 male, 12 female) Group 2: n: 17, Ricketts retractor. Retrospective study (4 ma, 13 fe)	Group 1: TMA 0.017 × 0.025 TLS. brackets: 0.022 in/edgewise Force: 344 g (at 6 mm activation) Group 2: blue Elgiloy 0.016 × 0.016 Ricketts maxillary canine retractor Brackets: 0.018 in/Ricketts prescription Force: 150 g.	Canine retraction efficacy (amount of canine retraction, tipping, rotation, torque, and root resorption) Anchorage loss control.	Until both canines reach a class I relationship	At the beginning of treatment. TPA	CBCT before retraction and after ending retraction of both left and right canines.

Y: years, m: Month, ma: Male, fe: Female, Slid M: Sliding Mechanics, Sect A: Sectional Archwire, CRs: Cephalometric Radiographs, CBCT: Cone-Beam Computed Tomography, PCT: Power Chain Traction, TLS: T-Loop Spring.

**Table 3:** Results of the included trials.

Authors	Rate of Canine Retraction	Tipping *	Rotation	Anchorage Loss & Root Resorption
Dincer and Iscan, 1994 [17]	Goup1: Right side (Reverse Closing Loop) Maxilla: 0.59 ± 0.35 mm/month. Mandible: 0.39 ± 0.15 mm/month Group2: Left side (Gjessing’s retraction spring) Maxilla: 0.85 ± 0.41 mm/month Mandible: 1.03 ± 0.85 mm/month	G1: 5.41 ± 5.18/7.75 months. G2: 3.33 ± 6.89/6.25 months. Nonsignificant		Anchorage loss: G1: 2.46 mm. G2: 1.63 mm.
Keng et al., 2012 [15]	T-loop (TMA): 0.87 ± 0.34 mm/month. T-loop (NITI): 0.91 ± 0.46 mm/month Nonsignificant	NITI: 0.71 ± 2.34°/month. TMA: 1.15 ± 2.86°/month. Nonsignificant		
Mehta and Sable, 2013 [18]	0.017 × 0.025 TMA T-loop: 5.46 mm/4 months 0.016 × 0.022 S.S. T-loop: 4.20 mm/4 months	TMA: 7.83°/4 months. S.S: 10°/4 months. Indicating that the TMA had better control.	S.S. T-loop offered better rotational control (39.44%) over the TMA T-loop (50.82%).	
Ozkan and Bayram, 2016 [16]	Direct anchorage: RCL: 1.57 ± 0.53 mm/4 weeks. LS: 1.80 ± 0.67 mm/4 weeks. Nonsignificant Indirect anchorage: RCL: 1.45 ± 0.69 mm/4 weeks. LS: 1.42 ± 0.63 mm/4 weeks Nonsignificant	Direct anchorage: RCL: 16.82 ± 9.19°/7.62 mm. LS: 16.24 ± 4.97°/7.62 mm. Nonsignificant Indirect anchorage: RCL: 15.66 ± 6.34°/7.25 mm. LS: 15.90 ± 5.12°/6.94 mm. Nonsignificant	Direct anchorage: RCL: 33.55°/7.62 mm LS: 33.71°/7.62 mm. Nonsignificant Indirect anchorage: RCL: 27.65°/7.25 mm LS: 28.19°/6.94 mm. Nonsignificant	Anchorage loss: Direct anchorage: RCL: 1.21 ± 2.48 mm LS: 0.02 ± 2.28 mm Nonsignificant Indirect anchorage: RCL: 1.01 ± 2.83 mm. LS: 0.91 ± 2.25 mm Nonsignificant
Davis et al., 2019 [14]	MS: 1.187 ± 0.232 mm/month. TLS: 0.708 ± 0.157 mm/month.	MS: 6.645 ± 2.744°/3 months. T-loop: 1.229 ± 5.124°/3 months.	MS: 2.416 ± 1.868°/3 months. T-loop: 5.645 ± 2.849°/3 months.	Anchorage loss: MS: 0.791 ± 0.142. T-loop: 0.250 ± 0.466.
Masaes et al., 2022 [5]	TLS: 1.85 ± 0.64 mm/5.92 ± 1.75 months. RMCR: 1.10 ± 0.63 mm/5.26 ± 1.10 months.	TLS: 8.06 ± 3.66°/5.92 ± 1.75 months. RMCR: 10.19 ± 3.99°/5.26 ± 1.10 months. Nonsignificant.	TLS: 5.71 ± 6.95 °/5.92 ± 1.75 months. RMCR: 14.99 ± 9.24 °/5.26 ± 1.10 months.	Anchorage loss: TLS: 1.40 ± 0.89 mm/5.92 ± 1.75 months. RMCR: 1.20 ± 0.65 mm/5.26 ± 1.10 months. Nonsignificant Root resorption: TLS: 0.51 ± 0.5 mm. RMCR: 0.62 ± 0.59 mm. Nonsignificant

*: The findings indicate how much tipping changed upon retraction, just as they did during the specified observation period. SM: Sliding Mechanics, SA: Sectional Archwire, NS: Not Significant, GRS: Gjessing’s Retraction Spring, RCRS: Ricketts Canine Retraction Spring, RMCR: Ricketts Maxillary Canine Retractor, RCL: Reverse Closing Loop, LS: Ladanyi Spring, MS: Modified Marcotte Spring, TLS: T-Loop Spring, NS: Nonsignificant, S: Statistically Significant.

## Data Availability

The corresponding authors may provide datasets and spreadsheets supporting the current work upon reasonable request.

## Appendix A

**Table A1:** Search strategy used in the current systematic review.

PubMed	#1 (Class II relationship OR extraction of the first premolars OR severe crowding OR maxillary dentoalveolar protrusion OR bimaxillary protrusion OR permanent occlusion OR anterior open bite) #2 (Upper anterior teeth retraction OR lower anterior teeth retraction OR space closure OR incisors retraction OR canine retraction OR En-masse retraction OR moving anterior teeth backward) #3 (Anchorage loss OR rotation OR inclination OR torque OR angulation OR tipping OR root resorption OR Orthodontic tooth movement rate OR orthodontic tooth movement amount OR orthodontic tooth movement velocity OR orthodontic tooth movement speed OR orthodontic tooth movement duration) #4 (Frictionless Mechanics OR Sectional Technique OR Segmental technique OR springs for retracting T-loop OR L-loop OR Ricketts's spring OR Marcotte spring OR Ladanyi spring OR Gjessing retraction spring OR Reverse Closing Loop) #5 #1 AND #2 AND #3 AND #4
CENTRAL (The Cochrane Library)	#1 (Class II relationship OR extraction of the first premolars OR severe crowding OR maxillary dentoalveolar protrusion OR bimaxillary protrusion OR permanent occlusion OR anterior open bite) #2 (Upper anterior teeth retraction OR lower anterior teeth retraction OR space closure OR incisors retraction OR canine retraction OR En-masse retraction OR moving anterior teeth backward) #3 (Anchorage loss OR rotation OR inclination OR torque OR angulation OR tipping OR root resorption OR Orthodontic tooth movement rate OR orthodontic tooth movement amount OR orthodontic tooth movement velocity OR orthodontic tooth movement speed OR orthodontic tooth movement duration) #4 (Frictionless Mechanics OR Sectional Technique OR Segmental technique OR springs for retracting T-loop OR L-loop OR Ricketts's spring OR Marcotte spring OR Ladanyi spring OR Gjessing retraction spring OR Reverse Closing Loop) #5 #1 AND #2 AND #3 AND #4
Web of Science	#1TS = (Class II relationship OR extraction of the first premolars OR severe crowding OR maxillary dentoalveolar protrusion OR bimaxillary protrusion OR permanent occlusion OR anterior open bite) #2TS = (Upper anterior teeth retraction OR lower anterior teeth retraction OR space closure OR incisors retraction OR canine retraction OR En-masse retraction OR moving anterior teeth backward) #3TS = (Anchorage loss OR rotation OR inclination OR torque OR angulation OR tipping OR root resorption OR Orthodontic tooth movement rate OR orthodontic tooth movement amount OR orthodontic tooth movement velocity OR orthodontic tooth movement speed OR orthodontic tooth movement duration) #4TS = (Frictionless Mechanics OR Sectional Technique OR Segmental technique OR springs for retracting T-loop OR L-loop OR Ricketts's spring OR Marcotte spring OR Ladanyi spring OR Gjessing retraction spring OR Reverse Closing Loop) #5 #1 AND #2 AND #3 AND #4
Scopus	#1 TITLE ABS (Class II relationship OR extraction of the first premolars OR severe crowding OR maxillary dentoalveolar protrusion OR bimaxillary protrusion OR permanent occlusion OR anterior open bite) #2 TITLE ABS-KEY (Upper anterior teeth retraction OR lower anterior teeth retraction OR space closure OR incisors retraction OR canine retraction OR En-masse retraction OR moving anterior teeth backward) #3 TITLE ABS-KEY (Anchorage loss OR rotation OR inclination OR torque OR angulation OR tipping OR root resorption OR Orthodontic tooth movement rate OR orthodontic tooth movement amount OR orthodontic tooth movement velocity OR orthodontic tooth movement speed OR orthodontic tooth movement duration) #4 TITLE ABS-KEY (Frictionless Mechanics OR Sectional Technique OR Segmental technique OR springs for retracting T-loop OR L-loop OR Ricketts's spring OR Marcotte spring OR Ladanyi spring OR Gjessing retraction spring OR Reverse Closing Loop) #5 #1 AND #2 AND #3 AND #4
EMBASE	#1 (Class II relationship OR extraction of the first premolars OR severe crowding OR maxillary dentoalveolar protrusion OR bimaxillary protrusion OR permanent occlusion OR anterior open bite) #2 (Upper anterior teeth retraction OR lower anterior teeth retraction OR space closure OR incisors retraction OR canine retraction OR En-masse retraction OR moving anterior teeth backward) #3 (Anchorage loss OR rotation OR inclination OR torque OR angulation OR tipping OR root resorption OR Orthodontic tooth movement rate OR orthodontic tooth movement amount OR orthodontic tooth movement velocity OR orthodontic tooth movement speed OR orthodontic tooth movement duration) #4 (Frictionless Mechanics OR Sectional Technique OR Segmental technique OR springs for retracting T-loop OR L-loop OR Ricketts's spring OR Marcotte spring OR Ladanyi spring OR Gjessing retraction spring OR Reverse Closing Loop) #5 #1 AND #2 AND #3 AND #4
Google scholar	(Class II relationship OR extraction of the first premolars OR severe crowding OR maxillary dentoalveolar protrusion OR bimaxillary protrusion OR permanent occlusion OR anterior open bite) AND (Upper anterior teeth retraction OR lower anterior teeth retraction OR space closure OR incisors retraction OR canine retraction OR En-masse retraction OR moving anterior teeth backward) AND (Anchorage loss OR rotation OR inclination OR torque OR angulation OR tipping OR root resorption OR Orthodontic tooth movement rate OR orthodontic tooth movement amount OR orthodontic tooth movement velocity OR orthodontic tooth movement speed OR orthodontic tooth movement duration) AND (Frictionless Mechanics OR Sectional Technique OR Segmental technique OR springs for retracting T-loop OR L-loop OR Ricketts's spring OR Marcotte spring OR Ladanyi spring OR Gjessing retraction spring OR Reverse Closing Loop)
Trip	(Class II relationship OR extraction of the first premolars OR severe crowding OR maxillary dentoalveolar protrusion OR bimaxillary protrusion OR permanent occlusion OR anterior open bite) AND (Upper anterior teeth retraction OR lower anterior teeth retraction OR space closure OR incisors retraction OR canine retraction OR En-masse retraction OR moving anterior teeth backward) AND (Anchorage loss OR rotation OR inclination OR torque OR angulation OR tipping OR root resorption OR Orthodontic tooth movement rate OR orthodontic tooth movement amount OR orthodontic tooth movement velocity OR orthodontic tooth movement speed OR orthodontic tooth movement duration) AND (Frictionless Mechanics OR Sectional Technique OR Segmental technique OR springs for retracting T-loop OR L-loop OR Ricketts's spring OR Marcotte spring OR Ladanyi spring OR Gjessing retraction spring OR Reverse Closing Loop)
